# (*E*)-2-{[(Furan-2-ylmeth­yl)imino]­meth­yl}-4-nitro­phenol

**DOI:** 10.1107/S1600536814005583

**Published:** 2014-03-15

**Authors:** Yousef Hijji, Samira Azemati, Ray J. Butcher, Jerry P. Jasinski

**Affiliations:** a Chemistry Department, Morgan State University, 1700 East Cold Spring Lane, Baltimore, MD 21251, USA; b Department of Chemistry, Howard University, 525 College Street NW, Washington, DC 20059, USA; c Department of Chemistry, Keene State College, Keene, NH 03410, USA

## Abstract

In the title compound, C_12_H_10_N_2_O_4_, the furan-2-ylmethyl group is disordered over two sets of sites, with refined occupancies of 0.858 (3) and 0.143 (3). In the major component of disorder, the dihedral angle between the furan and benzene rings is 63.1 (2)° and for the minor component this value is 67.9 (6)°. The planes of the nitro group and the attached benzene ring form a dihedral angle of 4.34 (17)°. In the crystal, inversion-related mol­ecules are linked by two pairs of weak C—H⋯O inter­actions, one involving the nitro group and the other involving the O—H group as an acceptor. As a result of these associations, ribbons are formed along [120]. A strong intra­molecular O—H⋯N hydrogen bond is observed.

## Related literature   

For the use of salicyl­idene compounds as anion sensors, see: Hijji *et al.* (2009 ▶) and for the use of related compounds as anion sensors, see: Hijji *et al.* (2004 ▶). For the bioactivity of metal complexes of structurally related salicyl­idene derivatives, see: Mandal *et al.* (2009*a* ▶,*b* ▶). For related structures, see: Song *et al.* (2008 ▶); Khalaji *et al.* (2011*a* ▶,*b* ▶).
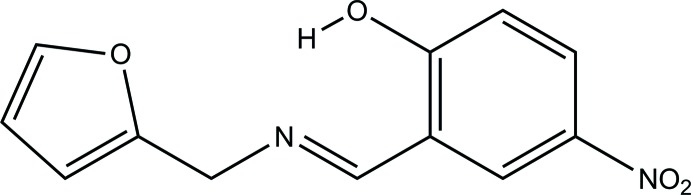


## Experimental   

### 

#### Crystal data  

C_12_H_10_N_2_O_4_
*M* *_r_* = 246.22
Triclinic, 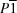

*a* = 5.4427 (7) Å
*b* = 8.2488 (10) Å
*c* = 12.4701 (14) Å
α = 98.901 (9)°
β = 92.04 (1)°
γ = 91.69 (1)°
*V* = 552.41 (12) Å^3^
*Z* = 2
Cu *K*α radiation
μ = 0.96 mm^−1^
*T* = 123 K
0.34 × 0.26 × 0.17 mm

#### Data collection  

Agilent Xcalibur (Ruby, Gemini) diffractometer
Absorption correction: multi-scan (*CrysAlis PRO*; Agilent, 2012 ▶) *T*_min_ = 0.912, *T*_max_ = 1.000
3400 measured reflections
2210 independent reflections
2047 reflections with *I* > 2σ(*I*)
*R*_int_ = 0.019

#### Refinement  

*R*[*F* ^2^ > 2σ(*F* ^2^)] = 0.042
*wR*(*F* ^2^) = 0.122
*S* = 1.06
2210 reflections
186 parameters
13 restraints
H atoms treated by a mixture of independent and constrained refinement
Δρ_max_ = 0.32 e Å^−3^
Δρ_min_ = −0.27 e Å^−3^

### 

Data collection: *CrysAlis PRO* (Agilent, 2012 ▶); cell refinement: *CrysAlis PRO*; data reduction: *CrysAlis PRO* (Agilent, 2012 ▶); program(s) used to solve structure: *SHELXS97* (Sheldrick, 2008 ▶); program(s) used to refine structure: *SHELXL2013* (Sheldrick, 2008 ▶); molecular graphics: *SHELXTL* (Sheldrick, 2008 ▶); software used to prepare material for publication: *SHELXTL*.

## Supplementary Material

Crystal structure: contains datablock(s) I. DOI: 10.1107/S1600536814005583/lh5694sup1.cif

Structure factors: contains datablock(s) I. DOI: 10.1107/S1600536814005583/lh5694Isup2.hkl

Supporting information file. DOI: 10.1107/S1600536814005583/lh5694Isup3.cml

CCDC reference: 991243

Additional supporting information:  crystallographic information; 3D view; checkCIF report

## Figures and Tables

**Table 1:** Hydrogen-bond geometry (Å, °)

*D*—H⋯*A*	*D*—H	H⋯*A*	*D*⋯*A*	*D*—H⋯*A*
O1—H1*O*⋯N2	0.95 (3)	1.72 (3)	2.5784 (14)	148 (2)
C2—H2*A*⋯O1^i^	0.95	2.52	3.4548 (16)	169
C7—H7*A*⋯O3^ii^	0.95	2.54	3.4567 (16)	161

Symmetry codes: (i) 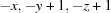; (ii) 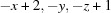.

**Fig. 1.**
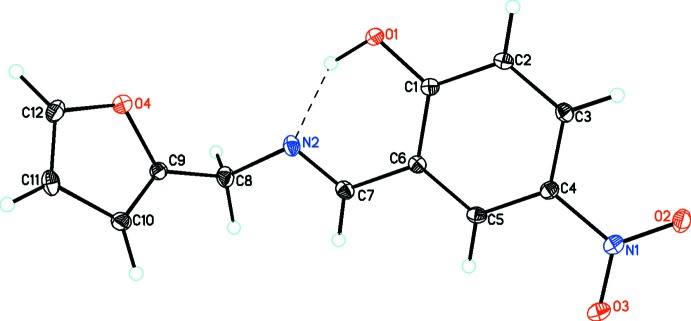
The molecular structure of the title compound showing ellipsoids at the 30% probabilty level (major component only). The dashed line indicates a hydrogen bond.

**Fig. 2.**
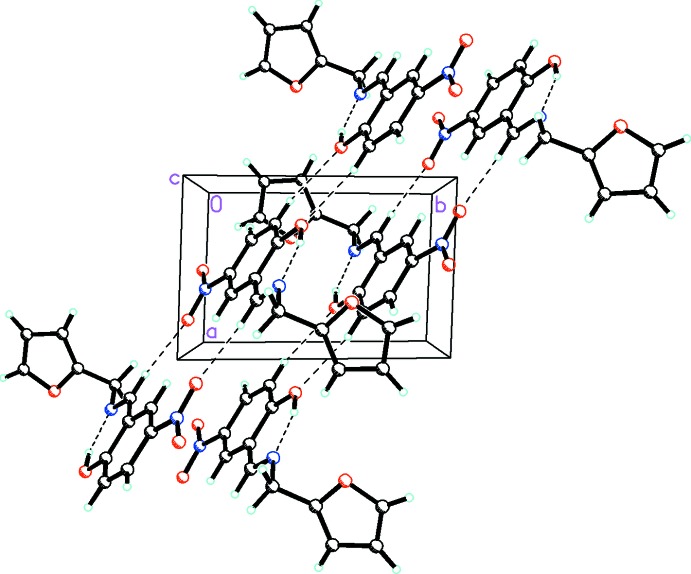
Packing diagram for the complex viewed along the c axis showing the repeating motif forming ribbons along [1 2 0]. N—H···O hydrogen bonds and C—H···O interactions shown by dashed lines.

## supplementary crystallographic information

### 1. Comment

The title compound adopts an E configuration with respect to the C═N imine bond. Structurally related salicyl­idene derivatives have been used as anion sensors (Hijji, *et al.*, 2004; Hijji *et al.*, 2009) and their metal complexes have shown bioactivity (Mandal *et al.*, 2009*a*,*b*). Similar structures have been previously reported (Song *et al.*, 2008; Khalaji *et al.*, 2011*a*,*b*).

The molecular structure of the title compound is shown in Fig. 1. The furan-2-yl­methyl group is disordered over two sets of sites with refined occupancies of 0.858 (3) and 0.142 (3). In the major component of disorder the dihedral angle between the furan and benzene rings is 63.1 (2)° and for the minor component this value is 67.9 (6)°. The nitro group and attached benzene ring form a dihedral angle of 4.34 (17)°. In the crystal, inversion related molecules are linked by two pairs of weak C—H···O inter­actions, one involves the nitro group while the other involves the O—H as an acceptor (Fig. 2). As a result of these associations ribbons are formed along [120]. A strong intra­molecular O—H···N hydrogen bond is observed.

### 2. Experimental

((E)-2-(((Furan-2-yl­methyl)­imino)­methyl)-4-nitro­phenol was synthesized by mixing 2-methyl­furyl­amine (0.29 g, 3mmol) with 5-nitro-2-hy­droxy­benzaldehyde. (0.32 g, 2 mmol) the mixture was mixed well and turned dark yellow. After mixing and grinding for 5 minutes the mixture was dissolved in 15 mL of di­ethyl ether and allowed to crystallize to give yellow crystals, 0.378g, 77% yield), m.p. (393-395 K). A sample was recrystallized from di­ethyl ether with slow evaporation to provide a crystal suitable for x-ray measurements.

### 2.1. Refinement

H atoms were placed in geometrically idealized positions with a C—H distances of 0.95 and 0.99 Å *U*ĩso~(H) = 1.2*U*~eq~(C) and 0.96 Å for CH~3~ [*U*ĩso~(H) = 1.5*U*~eq~(C)]. The furan ring was refined as disordered over two conformations. Both components were constrained to have similar geometries with occupancies of 0.858 (3) and 0.142 (3). For the hy­droxy group the H atom was refined isotropically.

### Crystal data

**Table:**

C_12_H_10_N_2_O_4_	*Z* = 2
*M**_r_* = 246.22	*F*(000) = 256
Triclinic, *P*1	*D*_x_ = 1.480 Mg m^−^^3^
*a* = 5.4427 (7) Å	Cu *K*α radiation, λ = 1.54178 Å
*b* = 8.2488 (10) Å	Cell parameters from 2639 reflections
*c* = 12.4701 (14) Å	θ = 3.6–75.5°
α = 98.901 (9)°	µ = 0.96 mm^−^^1^
β = 92.04 (1)°	*T* = 123 K
γ = 91.69 (1)°	Prism, pale yellow
*V* = 552.41 (12) Å^3^	0.34 × 0.26 × 0.17 mm

### Data collection

**Table:**

Agilent Xcalibur (Ruby, Gemini) diffractometer	2047 reflections with *I* > 2σ(*I*)
Detector resolution: 10.5081 pixels mm^-1^	*R*_int_ = 0.019
ω scans	θ_max_ = 75.6°, θ_min_ = 3.6°
Absorption correction: multi-scan (*CrysAlis PRO*; Agilent, 2012)	*h* = −6→6
*T*_min_ = 0.912, *T*_max_ = 1.000	*k* = −10→10
3400 measured reflections	*l* = −11→15
2210 independent reflections	

### Refinement

**Table:**

Refinement on *F*^2^	Primary atom site location: structure-invariant direct methods
Least-squares matrix: full	Secondary atom site location: difference Fourier map
*R*[*F*^2^ > 2σ(*F*^2^)] = 0.042	Hydrogen site location: inferred from neighbouring sites
*wR*(*F*^2^) = 0.122	H atoms treated by a mixture of independent and constrained refinement
*S* = 1.06	*w* = 1/[σ^2^(*F*_o_^2^) + (0.0764*P*)^2^ + 0.0922*P*] where *P* = (*F*_o_^2^ + 2*F*_c_^2^)/3
2210 reflections	(Δ/σ)_max_ < 0.001
186 parameters	Δρ_max_ = 0.32 e Å^−^^3^
13 restraints	Δρ_min_ = −0.27 e Å^−^^3^

### Special details

**Table:**

Experimental. CrysAlisPro, Agilent Technologies, Version 1.171.35.21 (release 20-01-2012 CrysAlis171 .NET) (compiled Jan 23 2012,18:06:46) Empirical absorption correction using spherical harmonics, implemented in SCALE3 ABSPACK scaling algorithm.^1^H-NMR (400 MHz): d ppm (CDCl_3_): 14.41(br. s 1H), 8.39 (t, J = 1.25 Hz, 1H) 8.255 (d, J = 2.85 Hz, 1H), 8.205 (dd, J = 8.25, 2.85 Hz, 1H), 7.44 ( dd, J = 1.75, 0.75 Hz, 1H), 7.01 ( d, J = 7.30 Hz, 1H), 6.395 (dd, J = 3.45, 1.85 Hz, 1H), 6.35 dt, J = 3.45, 0.75 Hz, 1 H), 4.84 (s, 2 H). ^13^C-NMR (100 MHz) d ppm (CDCl_3_): 167.64, 164.96, 149.57, 143.12, 139.45, 128.23, 128.08, 118.46, 117.44, 110.05, 108.99, 54.01. Mass spec: M^+^ = 246.
Geometry. All e.s.d.'s (except the e.s.d. in the dihedral angle between two l.s. planes) are estimated using the full covariance matrix. The cell e.s.d.'s are taken into account individually in the estimation of e.s.d.'s in distances, angles and torsion angles; correlations between e.s.d.'s in cell parameters are only used when they are defined by crystal symmetry. An approximate (isotropic) treatment of cell e.s.d.'s is used for estimating e.s.d.'s involving l.s. planes.

### Fractional atomic coordinates and isotropic or equivalent isotropic displacement parameters (Å^2^)

**Table:**

	*x*	*y*	*z*	*U*_iso_*/*U*_eq_	Occ. (<1)
O1	0.24922 (16)	0.42490 (11)	0.60178 (7)	0.0282 (2)	
H1O	0.352 (5)	0.424 (3)	0.665 (2)	0.082 (8)*	
O2	0.51829 (18)	0.00193 (12)	0.16611 (7)	0.0345 (2)	
O3	0.83082 (19)	−0.04412 (13)	0.26772 (8)	0.0406 (3)	
N1	0.6361 (2)	0.02267 (13)	0.25331 (9)	0.0281 (2)	
N2	0.6077 (2)	0.34958 (13)	0.72346 (8)	0.0281 (2)	
C1	0.3478 (2)	0.33032 (14)	0.51862 (9)	0.0226 (2)	
C2	0.2310 (2)	0.31430 (15)	0.41515 (10)	0.0244 (2)	
H2A	0.0863	0.3727	0.4049	0.029*	
C3	0.3249 (2)	0.21438 (14)	0.32834 (9)	0.0242 (2)	
H3A	0.2450	0.2024	0.2585	0.029*	
C4	0.5397 (2)	0.13099 (14)	0.34469 (9)	0.0233 (2)	
C5	0.6607 (2)	0.14649 (14)	0.44537 (10)	0.0240 (2)	
H5A	0.8072	0.0893	0.4542	0.029*	
C6	0.5667 (2)	0.24627 (14)	0.53369 (9)	0.0222 (2)	
C7	0.6911 (2)	0.26050 (14)	0.64088 (10)	0.0256 (3)	
H7A	0.8368	0.2022	0.6491	0.031*	
O4	0.6949 (2)	0.62761 (19)	0.92624 (12)	0.0322 (3)	0.858 (3)
C8	0.7459 (4)	0.3535 (3)	0.8281 (2)	0.0320 (5)	0.858 (3)
H8A	0.8813	0.2757	0.8180	0.038*	0.858 (3)
H8B	0.6350	0.3170	0.8816	0.038*	0.858 (3)
C9	0.8503 (5)	0.5208 (3)	0.8716 (3)	0.0253 (3)	0.858 (3)
C10	1.0759 (3)	0.5895 (2)	0.87026 (14)	0.0297 (4)	0.858 (3)
H10A	1.2147	0.5405	0.8362	0.036*	0.858 (3)
C11	1.0658 (4)	0.7526 (2)	0.93068 (14)	0.0318 (4)	0.858 (3)
H11A	1.1970	0.8325	0.9449	0.038*	0.858 (3)
C12	0.8342 (4)	0.76926 (19)	0.96289 (13)	0.0328 (4)	0.858 (3)
H12A	0.7744	0.8649	1.0049	0.039*	0.858 (3)
O4A	0.7528 (18)	0.6486 (14)	0.9445 (9)	0.0322 (3)	0.142 (3)
C8A	0.694 (3)	0.3590 (19)	0.8374 (16)	0.0320 (5)	0.142 (3)
H8A1	0.8057	0.2681	0.8444	0.038*	0.142 (3)
H8A2	0.5526	0.3481	0.8836	0.038*	0.142 (3)
C9A	0.827 (3)	0.519 (2)	0.8736 (17)	0.0253 (3)	0.142 (3)
C10A	1.0441 (18)	0.5434 (14)	0.8316 (9)	0.0297 (4)	0.142 (3)
H10B	1.1253	0.4736	0.7772	0.036*	0.142 (3)
C11A	1.1230 (19)	0.6970 (14)	0.8873 (9)	0.0318 (4)	0.142 (3)
H11B	1.2789	0.7477	0.8793	0.038*	0.142 (3)
C12A	0.950 (3)	0.7651 (13)	0.9539 (9)	0.0328 (4)	0.142 (3)
H12B	0.9601	0.8700	0.9981	0.039*	0.142 (3)

### Atomic displacement parameters (Å^2^)

**Table:**

	*U*^11^	*U*^22^	*U*^33^	*U*^12^	*U*^13^	*U*^23^
O1	0.0281 (4)	0.0311 (4)	0.0245 (4)	0.0086 (3)	0.0018 (3)	0.0001 (3)
O2	0.0409 (5)	0.0361 (5)	0.0247 (4)	0.0025 (4)	0.0013 (4)	−0.0014 (4)
O3	0.0365 (5)	0.0429 (6)	0.0409 (5)	0.0172 (4)	0.0048 (4)	−0.0024 (4)
N1	0.0291 (5)	0.0253 (5)	0.0298 (5)	0.0021 (4)	0.0052 (4)	0.0034 (4)
N2	0.0347 (5)	0.0247 (5)	0.0245 (5)	0.0003 (4)	−0.0056 (4)	0.0040 (4)
C1	0.0220 (5)	0.0210 (5)	0.0252 (5)	0.0008 (4)	0.0021 (4)	0.0048 (4)
C2	0.0205 (5)	0.0249 (5)	0.0283 (6)	0.0036 (4)	−0.0002 (4)	0.0059 (4)
C3	0.0243 (5)	0.0261 (5)	0.0229 (5)	−0.0002 (4)	−0.0010 (4)	0.0063 (4)
C4	0.0246 (5)	0.0203 (5)	0.0249 (6)	0.0009 (4)	0.0038 (4)	0.0027 (4)
C5	0.0212 (5)	0.0209 (5)	0.0308 (6)	0.0030 (4)	0.0016 (4)	0.0067 (4)
C6	0.0225 (5)	0.0202 (5)	0.0244 (5)	0.0003 (4)	−0.0007 (4)	0.0054 (4)
C7	0.0263 (5)	0.0211 (5)	0.0299 (6)	0.0011 (4)	−0.0047 (4)	0.0064 (4)
O4	0.0284 (7)	0.0340 (6)	0.0326 (7)	0.0038 (5)	0.0018 (5)	−0.0002 (5)
C8	0.0410 (13)	0.0279 (6)	0.0266 (8)	0.0046 (8)	−0.0087 (8)	0.0042 (5)
C9	0.0277 (8)	0.0287 (6)	0.0195 (5)	0.0067 (5)	−0.0021 (5)	0.0030 (4)
C10	0.0246 (7)	0.0411 (10)	0.0230 (8)	0.0050 (6)	0.0016 (6)	0.0032 (7)
C11	0.0386 (9)	0.0338 (9)	0.0222 (8)	−0.0078 (7)	−0.0049 (7)	0.0047 (6)
C12	0.0441 (10)	0.0261 (7)	0.0268 (7)	0.0069 (7)	−0.0003 (7)	−0.0011 (5)
O4A	0.0284 (7)	0.0340 (6)	0.0326 (7)	0.0038 (5)	0.0018 (5)	−0.0002 (5)
C8A	0.0410 (13)	0.0279 (6)	0.0266 (8)	0.0046 (8)	−0.0087 (8)	0.0042 (5)
C9A	0.0277 (8)	0.0287 (6)	0.0195 (5)	0.0067 (5)	−0.0021 (5)	0.0030 (4)
C10A	0.0246 (7)	0.0411 (10)	0.0230 (8)	0.0050 (6)	0.0016 (6)	0.0032 (7)
C11A	0.0386 (9)	0.0338 (9)	0.0222 (8)	−0.0078 (7)	−0.0049 (7)	0.0047 (6)
C12A	0.0441 (10)	0.0261 (7)	0.0268 (7)	0.0069 (7)	−0.0003 (7)	−0.0011 (5)

### Geometric parameters (Å, º)

**Table:**

O1—C1	1.3363 (14)	C8—C9	1.491 (3)
O1—H1O	0.95 (3)	C8—H8A	0.9900
O2—N1	1.2286 (15)	C8—H8B	0.9900
O3—N1	1.2289 (15)	C9—C10	1.339 (3)
N1—C4	1.4586 (15)	C10—C11	1.440 (3)
N2—C7	1.2747 (17)	C10—H10A	0.9500
N2—C8A	1.47 (2)	C11—C12	1.341 (3)
N2—C8	1.479 (3)	C11—H11A	0.9500
C1—C2	1.4036 (16)	C12—H12A	0.9500
C1—C6	1.4172 (16)	O4A—C9A	1.359 (14)
C2—C3	1.3788 (17)	O4A—C12A	1.410 (14)
C2—H2A	0.9500	C8A—C9A	1.483 (14)
C3—C4	1.3982 (17)	C8A—H8A1	0.9900
C3—H3A	0.9500	C8A—H8A2	0.9900
C4—C5	1.3830 (17)	C9A—C10A	1.333 (14)
C5—C6	1.3917 (17)	C10A—C11A	1.395 (12)
C5—H5A	0.9500	C10A—H10B	0.9500
C6—C7	1.4633 (16)	C11A—C12A	1.354 (13)
C7—H7A	0.9500	C11A—H11B	0.9500
O4—C9	1.362 (3)	C12A—H12B	0.9500
O4—C12	1.381 (2)		
			
C1—O1—H1O	108.3 (17)	H8A—C8—H8B	107.9
O2—N1—O3	123.29 (11)	C10—C9—O4	111.09 (19)
O2—N1—C4	118.49 (10)	C10—C9—C8	132.3 (2)
O3—N1—C4	118.22 (11)	O4—C9—C8	116.55 (19)
C7—N2—C8A	127.0 (7)	C9—C10—C11	106.24 (16)
C7—N2—C8	116.83 (12)	C9—C10—H10A	126.9
O1—C1—C2	119.08 (10)	C11—C10—H10A	126.9
O1—C1—C6	120.99 (10)	C12—C11—C10	106.29 (14)
C2—C1—C6	119.92 (11)	C12—C11—H11A	126.9
C3—C2—C1	120.40 (11)	C10—C11—H11A	126.9
C3—C2—H2A	119.8	C11—C12—O4	110.34 (14)
C1—C2—H2A	119.8	C11—C12—H12A	124.8
C2—C3—C4	119.02 (11)	O4—C12—H12A	124.8
C2—C3—H3A	120.5	C9A—O4A—C12A	104.9 (10)
C4—C3—H3A	120.5	N2—C8A—C9A	109.4 (15)
C5—C4—C3	121.81 (11)	N2—C8A—H8A1	109.8
C5—C4—N1	119.21 (10)	C9A—C8A—H8A1	109.8
C3—C4—N1	118.97 (11)	N2—C8A—H8A2	109.8
C4—C5—C6	119.65 (11)	C9A—C8A—H8A2	109.8
C4—C5—H5A	120.2	H8A1—C8A—H8A2	108.2
C6—C5—H5A	120.2	C10A—C9A—O4A	114.2 (11)
C5—C6—C1	119.19 (11)	C10A—C9A—C8A	117.8 (13)
C5—C6—C7	119.90 (10)	O4A—C9A—C8A	128.0 (13)
C1—C6—C7	120.90 (11)	C9A—C10A—C11A	102.9 (10)
N2—C7—C6	121.28 (11)	C9A—C10A—H10B	128.6
N2—C7—H7A	119.4	C11A—C10A—H10B	128.6
C6—C7—H7A	119.4	C12A—C11A—C10A	111.8 (9)
C9—O4—C12	106.02 (16)	C12A—C11A—H11B	124.1
N2—C8—C9	112.0 (2)	C10A—C11A—H11B	124.1
N2—C8—H8A	109.2	C11A—C12A—O4A	106.0 (9)
C9—C8—H8A	109.2	C11A—C12A—H12B	127.0
N2—C8—H8B	109.2	O4A—C12A—H12B	127.0
C9—C8—H8B	109.2		
			
O1—C1—C2—C3	−178.40 (10)	C8A—N2—C8—C9	−94 (3)
C6—C1—C2—C3	1.40 (18)	C12—O4—C9—C10	−1.4 (3)
C1—C2—C3—C4	−0.77 (18)	C12—O4—C9—C8	177.1 (3)
C2—C3—C4—C5	−0.24 (18)	N2—C8—C9—C10	−100.0 (4)
C2—C3—C4—N1	178.74 (10)	N2—C8—C9—O4	81.9 (3)
O2—N1—C4—C5	175.41 (10)	O4—C9—C10—C11	1.2 (3)
O3—N1—C4—C5	−4.15 (17)	C8—C9—C10—C11	−177.0 (4)
O2—N1—C4—C3	−3.60 (17)	C9—C10—C11—C12	−0.4 (2)
O3—N1—C4—C3	176.84 (11)	C10—C11—C12—O4	−0.44 (18)
C3—C4—C5—C6	0.62 (18)	C9—O4—C12—C11	1.1 (2)
N1—C4—C5—C6	−178.37 (10)	C7—N2—C8A—C9A	108.4 (12)
C4—C5—C6—C1	0.02 (17)	C8—N2—C8A—C9A	74 (3)
C4—C5—C6—C7	178.74 (10)	C12A—O4A—C9A—C10A	−4 (2)
O1—C1—C6—C5	178.78 (10)	C12A—O4A—C9A—C8A	177 (2)
C2—C1—C6—C5	−1.01 (17)	N2—C8A—C9A—C10A	−70 (2)
O1—C1—C6—C7	0.07 (17)	N2—C8A—C9A—O4A	109 (2)
C2—C1—C6—C7	−179.72 (10)	O4A—C9A—C10A—C11A	5 (2)
C8A—N2—C7—C6	171.7 (8)	C8A—C9A—C10A—C11A	−176.2 (18)
C8—N2—C7—C6	179.22 (13)	C9A—C10A—C11A—C12A	−4.2 (16)
C5—C6—C7—N2	−179.25 (11)	C10A—C11A—C12A—O4A	2.1 (13)
C1—C6—C7—N2	−0.55 (18)	C9A—O4A—C12A—C11A	0.9 (16)
C7—N2—C8—C9	116.69 (17)		

### Hydrogen-bond geometry (Å, º)

**Table:**

*D*—H···*A*	*D*—H	H···*A*	*D*···*A*	*D*—H···*A*
O1—H1*O*···N2	0.95 (3)	1.72 (3)	2.5784 (14)	148 (2)
C2—H2*A*···O1^i^	0.95	2.52	3.4548 (16)	169
C7—H7*A*···O3^ii^	0.95	2.54	3.4567 (16)	161

Symmetry codes: (i) −*x*, −*y*+1, −*z*+1; (ii) −*x*+2, −*y*, −*z*+1.
